# Supplementary material for: Splice-Junction-Based Mapping of Alternative Isoforms in the Human Proteome
Source: Cell Rep. Author manuscript; Available in PMC 2020 Jan 15. (PMC6961840; doi:10.1016/j.celrep.2019.11.026)
Supplement: 2 [file NIHMS1546469-supplement-2.zip › DF1/PXD006675/CardiacFibroblast/CardiacFibroblast_26_DPH7_VTAQGPALGAHTSPR.pdf]

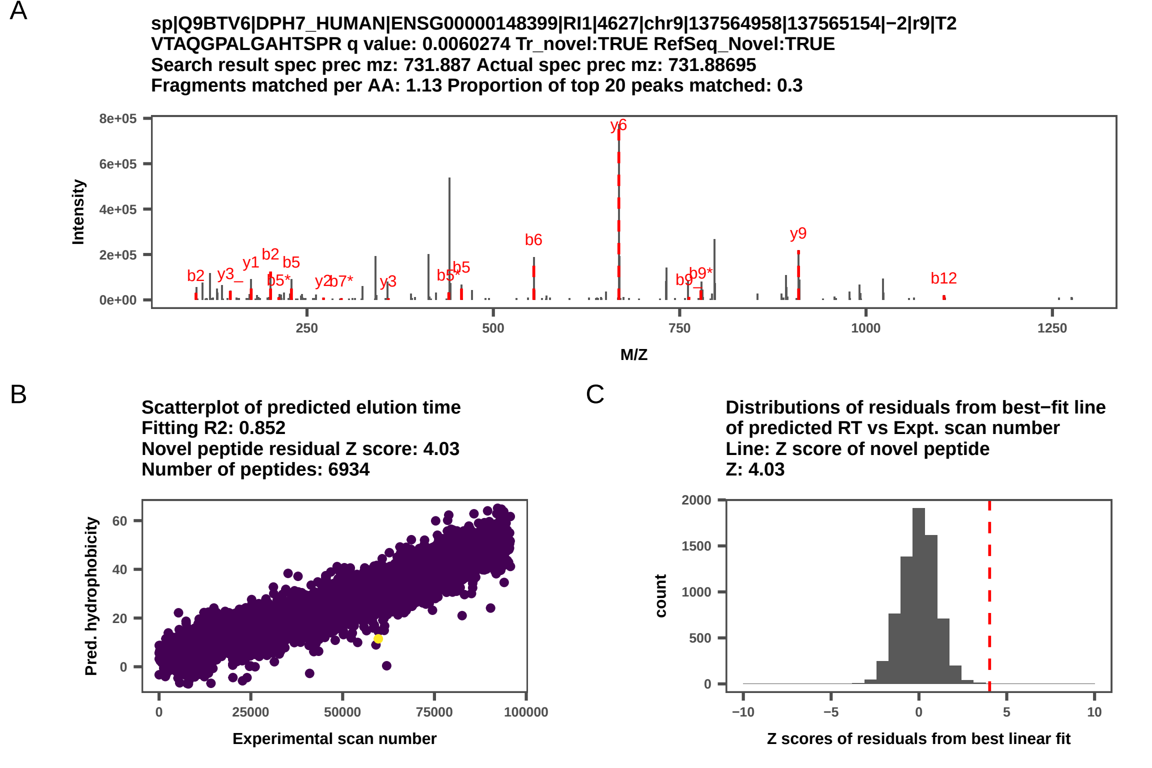
A
sp|Q9BTV6|DPH7_HUMAN|ENSG00000148399|RI1|4627|chr9|137564958|137565154|−2|r9|T2
VTAQGPALGAHTSPR q value: 0.0060274 Tr_novel:TRUE RefSeq_Novel:TRUE
Search result spec prec mz: 731.887 Actual spec prec mz: 731.88695
Fragments matched per AA: 1.13 Proportion of top 20 peaks matched: 0.3
Intensity
8e+05
6e+05
4e+05
2e+05
0e+00
y6
y9
b6
b12
b2
y3_
y1
b2
b5
b5*
y2
b7*
y3
b5*
b5
b9_
b9*
250
500
750
1000
1250
M/Z
B
Scatterplot of predicted elution time
Fitting R2: 0.852
Novel peptide residual Z score: 4.03
Number of peptides: 6934
Pred. hydrophobicity
60
40
20
0
0
25000
50000
75000
100000
Experimental scan number
C
Distributions of residuals from best−fit line
of predicted RT vs Expt. scan number
Line: Z score of novel peptide
Z: 4.03
count
2000
1500
1000
500
0
−10
−5
0
5
10
Z scores of residuals from best linear fit
